# Human colonic intraepithelial lymphocytes regulate the cytokines produced by lamina propria mononuclear cells

**DOI:** 10.1080/09629359791794

**Published:** 1997-04

**Authors:** P. Hoang, J. P. Dehennin, Li Li, C. Sibille, A. Geubel, J. P. Vaerman

**Affiliations:** 1 Department of Gastroenterology University Hospital St-Luc Brussels B-1200 Belgium; 2 Department of Medical Genetics Loverval Belgium; 3 Experimental Medicine Unit Institute of Cell Pathology Brussels B-1200 Belgium

## Abstract

Using an *in vitro* autologous human system, the immunomodulatory function of colonic intraepithelial lymphocytes (IEL) on cytokine production by lamina propria mononuclear cells (LPMNC) has been investigated. In contrast to LPMNC, colonic IEL produced only low amounts of IL-10, interferon-γ and interleukin-2. However, co-culture experiments (IEL + LPMNC) have shown that IEL can enhance the PHA-induced synthesis of IL-2 and interferon-γ, but not IL-10 by LPMNC. Using a transwell filter culture system apparatus, this effect was shown not to require a cell-to-cell interaction. Thus, IEL *in vitro* may modulate the cytokine synthesis of LPMNC, through the production of soluble factors. This may prove highly relevant in the *in vivo* immune activation of the gastrointestinal mucosa.


Mediators of Inflammation, 6, 105–109 (1997)

### Human colonic intraepithelial lymphocytes regulate the cytokines produced by lamina propria mononuclear cells

P. Hoang,[1,CA] J. P. Dehennin,[1] Li Li,[1] C. Sibille,[2] A. Geubel[1] and J. P. Vaerman[3]

[1]Department of Gastroenterology, University Hospital St-Luc, B-1200 Brussels; [2]Department of Medical Genetics, Loverval; [3]Experimental Medicine Unit, Institute of Cell Pathology, B-1200 Brussels, Belgium

[CA]Corresponding Author
Fax: (+32) 2 7648927

**USING an *in vitro* autologous human system, the immunomodulatory function of colonic intraepithelial lymphocytes (IEL) on cytokine production by lamina propria mononuclear cells (LPMNC) has been investigated. In contrast to LPMNC, colonic IEL produced only low amounts of IL-10, interferon-γ and interleukin-2. However, co-culture experiments (IEL + LPMNC) have shown that IEL can enhance the PHA-induced synthesis of IL-2 and interferon-γ, but not IL-10 by LPMNC. Using a transwell filter culture system apparatus, this effect was shown not to require a cell-to-cell interaction. Thus, IEL *in vitro* may modulate the cytokine synthesis of LPMNC, through the production of soluble factors. This may prove highly relevant in the *in vivo* immune activation of the gastrointestinal mucosa.**

**Key words:** Interferon-γ, Interleukin-2, Interleukin-10, Intraepithelial lymphocytes

### Introduction

Because of their unique location within the epithelium, their peculiar morphological and phenotypic features (different from those of peripheral blood and lamina propria lymphocytes) and their oligoclonal expression of αβ T cell receptors, many studies have focused on IEL function. Although their precise role *in vivo* remains enigmatic, it has been shown *in vitro* that, under certain conditions human IEL (hIEL) exert various patterns of cytotoxicity, are capable of proliferation, and perform some regulatory functions.[1–9] It is believed that they may interact with the adjacent epithelial cells and underlying lamina propria cells. In particular, we have previously shown that colonic hIEL can inhibit the proliferation of autologous LPL.[10] Moreover, hIEL were also able to suppress IgA synthesis.[11] Inhibitory mechanisms were shown to occur through soluble factors, possibly cytokines.

While some cytokines have a critical role in inducing inflammation, others are crucial in reducing inflammation and mediating the healing process. Most studies on murine IEL (mIEL) have used lymphocytes from the small intestine. Freshly isolated mIEL produce interferon-γ, and IL-5.[12] Subsequently, it has been shown that both TCR-αβ and γδ mIEL could produce interferon-γ, IL-2, IL-3 and IL-6 but not IL-4.[13,14] However, the amounts of cytokines released by γδ mIEL were much lower compared with αβ mIEL. In addition, culture supernatants from anti-TCR antibody-activated γδ or αβ mIEL trigger synthesis of TNF-α, TGF-β and GM-CSF that influence epithelial function.[14–16] Murine colonic IEL expressed similar levels of IL-1, interferon-γ and TNF-α mRNA, and significantly more IL-2, IL-4, IL-5 and IL-10 mRNA when compared with small intestine mIEL.[17] Recently, human IEL were analysed for cytokine mRNA expression: freshly isolated hIEL expressed high levels of IL-1β, IL-8, IL-2R and IFN-γ mRNA, but significantly less IL-1α, IL-6, IL-7, TNF-α and TNF-β mRNA. After *in vitro* polyclonal activation with phytohaemagglutinin (PHA) and PMA, IL-2 and IFN-γ mRNA were significantly increased, whereas IL-1 mRNA was significantly decreased.[18,19] Furthermore, hIEL can produce small amounts of IL-2 and IFN-γ in response to PHA even though there is little proliferation.[1,6,20] The production of these cytokines by hIEL is markedly increased by addition of sheep red blood cells to PHA or after stimulation through the CD2 molecule.[1] In hIEL, PHA induced no IL-

© 1997 Rapid Science Publishers

4 production while superantigen stimulation resulted in detectable IL-4.[9,21]

It is thus reasonable to consider the possibility that IEL may influence LPL function through the secretion of soluble factors. Therefore, colonic hIEL were tested for their ability to produce IL-2, IFN-γ and IL-10. Furthermore, the putative immunoregulatory function of IEL on cytokine secretion by autologous LPL was examined.

### Materials and Methods

### Subjects

Normal mucosa from colonic specimens was used from 10 control patients who were undergoing resection for a colonic carcinoma. The specimens were taken at least 5 cm away from any macroscopic lesion.

### Isolation of mucosal lymphocytes

IEL were isolated as previously described.[10] Briefly, to isolate colonic IEL, mucosal strips were immersed in RPMI-supplemented with fetal calf serum (FCS), antibiotics (gentamycin 50 μg/ml, penicillin 100 U/ml, streptomycin 100 μg/ml). The washed specimens were then stirred in the same medium with the addition of 1 mM dithiothreitol (Sigma) for 10 min. After further washing, they were then incubated with calcium- and magnesium-free HBSS and 0.75 mM ethylenediaminetetraacetate (EDTA) in a shaking water bath at 37°C for three periods of 30 min each at 140 oscillations per minute. This step was repeated twice and the resulting supernatants were stored overnight at 4°C. Further purification was achieved by passing this crude preparation down a glass wool column. The filtrate was then centrifuged at 800 ***g*** for 30 min through a discontinuous 44/67.5% Percoll bilayer. The IEL were recovered from the interface. To isolate the lamina propria mononuclear cells (LPMNC), the treated tissue was submitted to three more 45-min incubations with calcium- and magnesium-free HBSS and 5 mM EDTA. The mucosal strips were digested overnight at 37°C with 100 ml RPMI 1640 supplemented as above plus 30 mg *Clostridium histolyticum* collagenase (Boehringer Mannheim, Germany). The digestate was passed through a 100 μm nylon mesh filter. After separation on Ficoll-paque gradient, the LPL were washed and resuspended in RPMI-supplemented as above. The viability of the cells was assessed using 0.1% trypan blue.

### Preparation of cytokine-containing cell culture supernatants hIEL or LPMNC at a concentration of $1 \times 10^6$ were cultured in 1 ml aliquots in 24-well plates with or without 10 μg/ml PHA for 72 h at 37°C in 5% $CO_2$. The supernatants were then harvested, centrifuged and passed through a 0.22 μm filter and stored at −20°C for subsequent cytokine analysis.

### Influence of IEL on the cytokine production by autologous LPL

LPMNC ($10^6$) were cultured with IEL ($10^6$) for 72 h with or without PHA (10 μg/ml) in 24-well plates in a final volume of 1 ml at 37°C in 5% $CO_2$. The control cultures consisted of IEL or LPMNC alone, with or without PHA. At the end of the culture, the supernatants were harvested, centrifuged, passed through a 0.22 μm filter and stored at − 20°C for subsequent cytokine analysis (IL-2, IFN-γ and IL-10). In addition, IEL were co-cultured with LPMNC in a Transwell apparatus (Costar Cambridge, MA) (6.5 mm diameter, 3 μm pore size) which physically separated IEL from LPMNC. The purpose of this series of experiments was to investigate whether the immunoregulatory function of IEL is mediated by a soluble factor or requires interaction between IEL and LPMNC.

### Cytokine assays

Gamma-interferon, interleukin-10 (IL-10) and interleukin-2 (IL-2) were measured by a sensitive enzyme amplified immunoassay (Medgenix Diagnostics, Fleurus, Belgium). Optical densities were measured by an automated dual beam ELISA reader at 450 nm for γ-IFN, IL-10 and IL-2. Concentrations of these cytokines were determined by reference to the serially diluted standards included in each plate. The minimum detectable concentrations of these assays were: γ-FN 0.03 IU/ml; IL-10 1 pg/ml; IL-2 0.1 IU/ml. In these assays, duplicate values varied by < 5% from their mean value.

### Results

### Cytokine production by colonic mucosal lymphocytes

The median concentrations of cytokines secreted by IEL and LPMNC alone were respectively 0.3 IU/ml (range 0–0.6) and 1.5 IU/ml (range 1–8) for IFN-γ, 1 IU/ml (range 0–20) and 2.1 IU/ml (range 0.8–2.4) for IL-2, 8 pg/ml

(range 1–18) and 17 pg/ml (range 3.5–39) for L-10. Following stimulation with PHA, the median IL-2 concentrations by control IEL and LPMNC cultured alone were respectively 2.5 IU/ml (range 1–26) and 9 IU/ml (range 2–450), the median IFN-γ levels were 27 IU/ml (range 1–105) and 26.5 U/ml (range 2–242), the median IL-10 concentrations 3 pg/ml (range 1–10) and 4 pg/ml (range 1–100).

### Influence of IEL on the cytokine production by autologous LPMNC

After 3 days of culture, the effect of IEL on cytokine synthesis in co-culture with autologous LPMNC was studied. Addition of $10^6$ IEL only minimally affected IL-10 production. The median secretion of IL-10 secreted by PHA-stimulated LPMNC was 4 pg/ml (range 1–100). Following the addition of IEL, the concentrations increased to 7.5 pg/ml (range 2–340). In contrast, they enhanced significantly the IL-2 and IFN-γ production by LPMNC. After 3 days of co-culture, IL-2 and IFN-γ levels were respectively 39.5 IU/ml (range 8–450) and 104 IU/ml (18–500) (Figs 1, 2 and 3). Using the Transwell culture apparatus, similar results were obtained (Table 1).

### Discussion

The present data confirm our previous work showing that colonic IEL produce very low amounts of interferon-γ.[20] Although there was a significant increase in interferon-γ production following stimulation with PHA, the absolute

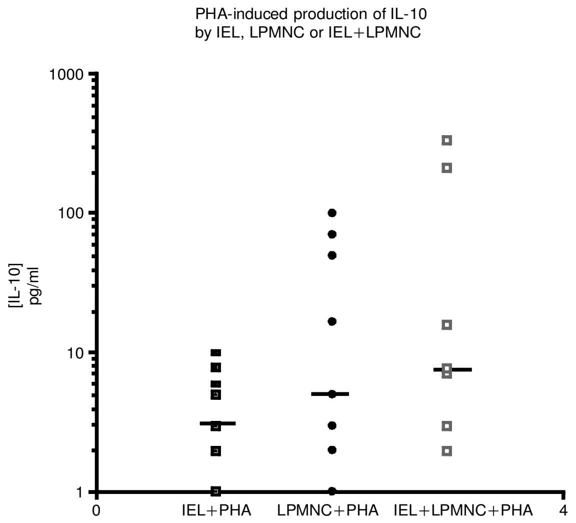

FIG. 1. Interleukin-10 concentration (pg/ml) secreted by PHA-stimulated mucosal lymphocytes IL-10 production by LPMNC is minimally affected by the addition of IEL.

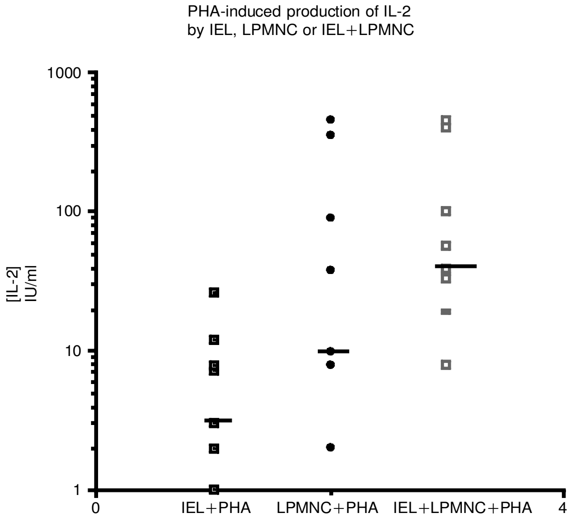

FIG. 2. Interleukin-2 concentration (IU/ml) secreted by PHA-stimulated mucosal lymphocytes. IL-2 production by LPMNC is significantly affected by the addition of IEL.

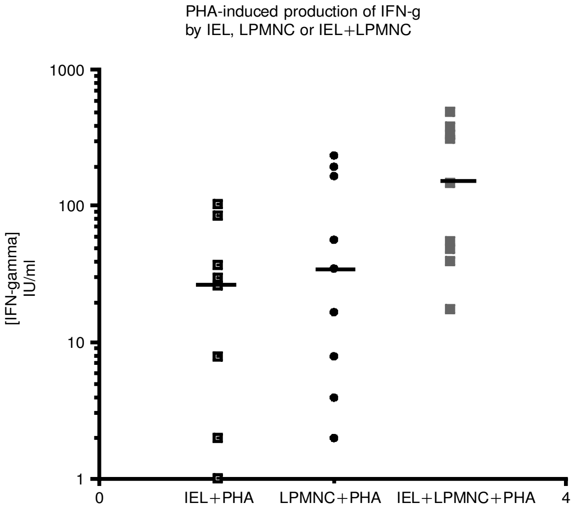

FIG. 3. Interferon-gamma concentration (IU/ml) secreted by mucosal lymphocytes. IFN-gamma secretion by LPMNC is significantly increased by the addition of IEL.

**Table 1**. Cytokine production by PHA-stimulated mucosal lymphocytes physically separated in a Transwell apparatus

| | IEL + LPMNC + PHA | IEL + LPMNC + PHA (transwell) |
|---|---|---|
| IL-10 (pg/ml) | 2.5 (2–6.7) | 1.9 (0.3–4.6) |
| IL-2 (IU/ml) | 31.8 (7.8–55.2) | 18.4 (4.2–70.8) |
| IFN-γ (IU/ml) | 49 (18–51) | 90 (6.6–158) |

Median (range) cytokines production, n = 5.

amounts of interferon-γ were persistently low. Sperber *et al.* reported similar findings using IEL stimulated with PHA or superantigens.[9] This was not due to the failure of lectin binding.[22] The amount of interferon-γ produced by stimulated hIEL was of sufficient quantity to induce MHC class II expression molecules on HT-29 cells.[20] Class II bearing epithelial cells can in turn cause further activation of hIEL.[23]

IL-2 is a central regulator of immune and inflammatory responses. It affects T cell proliferation as well as B cells and the function of macrophages, natural killer cells and lymphokine activated killer cell. PHA-stimulated colonic hIEL produce similar low amounts of interleukin-2, as do PHA-stimulated jejunal hIEL.[6,20] Conversely, Sperber *et al.* showed that IL-2 production was not observed with PHA- or superantigen-stimulated IEL.[9] Interestingly, induction of low levels of IL-2 does not correlate with the induction of *in vitro* proliferation. However, addition of exogeneous IL-2 does not restore a normal proliferative response.[6]

IL-10 is a powerful suppressor of cytokines produced by macrophages and Th2 subpopulations of $CD4^+$ T cells.[24] In addition, IL-10 inhibits antigen presentation but supports B cell differentiation and IgA secretion.[25,26] To the best of our knowledge, this is the first observation that human colonic IEL produce small amounts of IL-10 (as do colonic lamina propria cells). However, the precise action of this cytokine in the intestinal mucosa remains to be defined although a colitis occurs in mice in which there has been targeted deletion of the IL-10 gene.[27] Teitelbaum *et al.* have shown that neutralizing antibody to IL-10 did not block the inhibitory activity of rat intestinal intraepithelial lymphocytes on the proliferative response of allogeneic spleen-thymus cells stimulated with concacanavalin A.[15]

Murine helper T cells can be classified in relation to their cytokine production profile. Thus, Th1 cells produce large quantities of IL-2, IFN-γ and TNF-β while Th2 cells produce preferentially IL-4, IL-5, IL-6 and IL-10.[24] It has been suggested that a subset of murine CD8 IEL can produce some type 1 and 2 cytokines.[12,13] Furthermore, it has been shown that some human $CD8^+$ clones may secrete both patterns of cytokines.[28,29] It thus appears that these two subsets (Th1 and Th2) are mutually antagonistic. Th1 cells may influence Th2 cell function through these cytokines and vice versa. For example, interferon-γ may be able to downregulate a Th2 response while IL-10 has powerful inhibitory effects of the synthesis of Th1 cytokines.

The crucial role of cytokines in the mucosal response is well illustrated by a number of animal models of inflammatory bowel disease. IL-2-, IL-10 and TGF-β1 knockout mice develop chronic intestinal inflammation.[27,30,31] The local functions of these cytokines secreted by colonic hIEL have not been fully investigated. The data presented in this paper indicate that colonic hIEL can enhance the PHA-induced synthesis of IL-2 and interferon-γ by human colonic LPMNC. The upregulated LPMNC cytokine synthesis in hIEL + LPMNC co-cultures revealed that IEL did not contain cells inhibiting synthesis of these cytokines. Furthermore, absolute amounts of IL-10 produced by PHA-stimulated hIEL and LPMNC cultured alone or in co-culture were persistently low. Thus, the increased release of the Th1-cytokines may prove of importance in the modulation of subsequent immunopathological reactions including both activation of inflammatory cells and expression of class II molecules on epithelial cells.

Additional work must be done to understand the hIEL inhibitory activity previously described.[10,11] As IL-4 is virtually not produced by hIEL, further studies must be done in order to determine other inhibitory cytokines produced by IEL such as TGF-β and other inhibitory factors (e.g. neuropeptides).

It is important to note that cytokines and neuropeptides might in turn influence hIEL function. It has been suggested that interleukin-7 (IL-7) might be produced in the mucosa by intestinal epithelial cells, especially globlet cells.[8] Interleukin-7 is a B- and T-cell growth factor. It affects the proliferation of activated but not unstimulated human T cells. Recent reports have shown that IL-7 stimulates hIEL proliferation.[7,8] In contrast, IL-9 or IL-12 did not stimulate the proliferation of IEL.[7] IL-4 secreted by activated lamina propria cells suppresses IEL proliferation and LAK activity.[21] Finally two neuropeptides, substance P and vasoactive intestinal peptide induce no proliferation nor spontaneous cytotoxicity by hIEL. This was due to an absence of substance P and vasoactive intestinal peptide receptors.[32,33]

In conclusion, human colonic IEL may influence the cytokines produced by LPMNC. This *in vitro* observation may be of importance in the local *in vivo* immune activation of the gastrointestinal tract.

### References

1. Ebert EC. Intra-epithelial lymphocytes: interferon-gamma production and suppressor/cytotoxic activities. *Clin Exp Immunol* 1990; **82**: 81–85.
2. Cerf-Bensussan N, Guy-Grand D, Griscelli C. Intraepithelial lymphocytes of human gut: isolation, characterisation and study of natural killer activity. *Gut* 1985; **26**: 81–88.
3. Ebert EC, Roberts AI. Lymphokine-activated killing by human intestinal lymphocytes. *Cell Immunol* 1993; **146**: 107–116.
4. Rüthlein J, Heinze G, Auer IO. Anti-CD2 and anti-CD3 induced T cell cytotoxicity of human intraepithelial and lamina propria lymphocytes. *Gut* 1992; **33**: 1626–1632.

5. Ebert EC. Proliferative responses of human intraepithelial lymphocytes to various T-cell stimuli. *Gastroenterology* 1989; **97**: 1372–1381.
6. Ebert EC, Roberts AI, Brolin RE, Raska K. Examination of the low proliferative capacity of human jejunal intraepithelial lymphocytes. *Clin Exp Immunol* 1986; **65**: 148–157.
7. Bilenker M, Roberts AI, Brolin RE, Ebert E. Interleukin-7 activates intestinal lymphocytes. *Dig Dis Sci* 1995; **40**: 1744–1749.
8. Watanabe M, Ueno Y, Yajima T, Iwao Y, Tsuchiya M, Ishikawa H, Aiso S, Hibi T, Ishii H. Interleukin 7 is produced by human intestinal epithelial cells and regulates the proliferation of intestinal mucosal lymphocytes. *J Clin Invest* 1995; **95**: 2945–2953.
9. Sperber K, Silverstein L, Brusco C, Yoon C, Mullin GE, Mayer L. Cytokine secretion induced by superantigens in peripheral blood mononuclear cells, lamina propria lymphocytes, and intraepithelial lymphocytes. *Clin Diagn Lab Immunol* 1995; **2**: 473–477.
10. Hoang P, Dalton HR, Jewell DP. Human colonic intra-epithelial lymphocytes are suppressor cells. *Clin Exp Immunol* 1991; **85**: 498–503.
11. Sachdev GK, Dalton HR, Hoang P, DiPaolo MC, Crotty B, Jewell DP. Human colonic intraepithelial lymphocytes suppress in vitro immunoglobulin synthesis by autologous peripheral blood lymphocytes and lamina propria lymphocytes. *Gut* 1993; **34**: 257–263.
12. Taguchi T, McGhee JR, Coffman RL, Beagley KW, Eldridge JH, Takatsu K, Kiyono H. Analysis of Th1 and Th2 cells in murine gut-associated tissues. Frequencies of $CD4^+$ and $CD8^+$ T cells that secrete IFN-$\gamma$ and IL-5. *J Immunol* 1990; **145**: 68–77.
13. Taguchi T, Aicher WK, Fujihashi K, Yamamoto M, McGhee JR, Bluestone JA, Kiyono H. Novel function for intestinal intraepithelial lymphocytes. Murine $CD3^+$, $\gamma\delta$ $TCR^+$ T cells produce IFN-$\gamma$ and IL-5. *J Immunol* 1991; **147**: 3736–3744.
14. Barrett TA, Gajewski TF, Danielpour D, Chang EB, Beagley KW, Bluestone JA. Differential function of intestinal intraepithelial lymphocyte subsets. *J Immunol* 1992; **149**: 1124–1130.
15. Teitelbaum DH, Neideck B, Lee J, Merion R. Inhibitory activity of intestinal intraepithelial lymphocytes. *Surgery* 1995; **118**: 378–384.
16. Dillon SB, Dalton BJ, MacDonald TT. Lymphokine production by mitogen and antigen activated mouse intraepithelial lymphocytes. *Cell Immunol* 1986; **103**: 326–338.
17. Beagley KW, Fujihashi K, Lagoo AS, Lagoo-Deenadaylan S, Black CA, Murray AM, Sharmanov AT, Yamamoto M, McGhee JR, Elson CO, Kiyono H. Differences in intraepithelial lymphocyte T cell subsets isolated from murine small versus large intestine. *J Immunol* 1995; **154**: 5611–5619.
18. Deusch K, Wagner F, Daum S, Gstettenbauer M, Wiessel A, Classen M. Lymphokine repertoire and proliferative capacity of human intestinal intraepithelial lymphocytes. *Gastroenterology* 1991; **100**: A574.
19. Lundqvist C, Baranov V, Söderström K, Athlin L, Kiessling R, Hammarström S, Hammarström ML. Phenotype and cytokine profile of intraepithelial lymphocytes in human small and large intestine. *Ann NY Acad Sci* 1995; **7**: 395–399.
20. Hoang P, Dalton HR, De Silva HJ, Jewell DP. Cytokine production by human colonic intraepithelial lymphocytes in controls and ulcerative colitis. *Mediators of Inflammation* 1994; **3**: 219–222.
21. Ebert EC, Roberts AI. IL-4 down-regulates the responsiveness of human intraepithelial lymphocytes. *Clin Exp Immunol* 1996; **105**: 556–560.
22. Greenwood JH, Austin LL, Dobbins III WO. In vitro characterization of human intestinal intraepithelial lymphocytes. *Gastroenterology* 1983; **85**: 1023–1035.
23. Hoang P, Crotty B, Dalton HR, Jewell DP. Epithelial cells bearing class II molecules stimulate allogeneic human colonic intraepithelial lymphocytes. *Gut* 1992; **33**: 1089–1093.
24. Mosmann TR, Moore KW. The role of IL-10 in cross regulation of Th1 and Th2 responses. *Immunol Today* 1991; **12**: A49–A53.
25. Ding L, Linsley PS, Huang LY, Germain RN, Shevach EM. IL-10 inhibits macrophage costimulatory activity by selectively inhibiting the up-regulation of B7 expression. *J Immunol* 1993; **151**: 1224–1234.
26. Defrance T, Vanbervliet B, Briere F, Durand I, Rousett, F Banchereau J. Interleukin 10 and TGF-$\beta$ cooperate to induce anti-CD40-activated naive human B cells to secrete immunoglobulin A. *J Exp Med* 1992; **175**: 671–682.
27. Kühn R, Löhler J, Rennick D, Rajewsky K, Müller W. Interleukin-10-deficient mice develop chronic enterocolitis. *Cell* 1993; **75**: 263–274.
28. Salgame PR, Abrams JS, Clayberger C, Goldstein H, Convit J, Modlin RL, Bloom BR. Differing lymphokine profiles of functional subsets of human CD4 and CD8 T cell clones. *Science* 1991; **254**: 279–282.
29. Bloom SR, Salgame P, Diamond P. Revisiting and revising suppressor T cells. *Immunol Today* 1992; **13**: 131–136.
30. Sadlack B, Merz H, Schorle H, Schimpl A, Feller AC, Horak I. Ulcerative colitis-like disease in mice with a disrupted interleukin-2 gene. *Cell* 1993; **75**: 253–261.
31. Kulkarni AB, Huh CG, Becker D, Geiser A, Lyght M, Flanders KC, Roberts AB, Sporn MB, Ward JM, Karlsson S. *Proc Natl Acad Sci USA* 1993; **90**: 770–774.
32. Roberts AI, Panja A, Brolin RE, Ebert EC. Human intraepithelial lymphocytes. Immunomodulation and receptor binding of vasoactive intestinal peptide. *Dig Dis Sci* 1991; **36**: 341–346.
33. Roberts AI, Taunk J, Ebert EC. Human lymphocytes lack substance P receptors. *Cell Immunol* 1992; **141**: 451–465.

ACKNOWLEDGEMENTS. P.H. was supported by the Fonds de la Recherche Scientifique Médicale, Brussels (no. 3.4541.92).

**Received 17 December 1996; accepted 12 February 1997**
